# Pharmacological heat-shock protein inducers and chemical chaperones inhibit upregulation of interleukin-8 by oxidized phospholipids

**DOI:** 10.1007/s10787-022-01124-6

**Published:** 2023-01-24

**Authors:** Klara Hellauer, Olga V. Oskolkova, Bernd Gesslbauer, Valery Bochkov

**Affiliations:** 1 Institute of Pharmaceutical Sciences, Department of Pharmaceutical Chemistry, University of Graz, Humboldtstrasse 46/III, 8010 Graz, Austria; 2 Field of Excellence BioHealth, University of Graz, Graz, Austria

**Keywords:** Heat-shock proteins, Heat-shock protein inducers, Chemical chaperones, Interleukin 8, Oxidised phospholipids

## Abstract

Oxidised phospholipids such as oxidised palmitoyl-arachidonoyl-phosphatidylcholine (OxPAPC) are increasingly recognised as danger-associated molecular patterns (DAMPs) inducing cyto- and chemokines. The pathological impact of oxidised phosphatidylcholine in vivo has been demonstrated in several animal models, as well as in human association studies. In this work, we have tested a number of small molecules with known or potential anti-inflammatory properties for their ability to inhibit secretion of interleukin-8 by OxPAPC-treated endothelial cells. Six compounds capable of inhibiting the induction of IL-8 were selected. Analysis of gene expression has shown that all these substances reduced the OxPAPC-induced elevation of IL-8 mRNA but potentiated induction of heat-shock proteins (HSPs). We further found that drug-like HSP inducers also prevented the induction of IL-8 by OxPAPC. Similar inhibitory action was demonstrated by two chemical chaperones, which stabilise proteins through physicochemical mechanisms thus mimicking effects of HSPs. Our data suggest that proteostatic stress plays an important mechanistic role in the pro-inflammatory effects of OxPAPC and that stabilisation of proteome by overexpression of HSPs or by chemical chaperones can reduce the pro-inflammatory effects of OxPLs.

## Introduction

Oxidised phospholipids (OxPLs), which are generated by enzymatic or non-enzymatic oxidation of esterified PUFAs, are increasingly recognised for their multiple biological activities differing from those of non-oxidised precursor PLs (Oskolkova and Bochkov 2020). OxPLs are pleiotropic biologically active lipids targeting multiple cell types including the endothelium, where they activate mechanisms potentially relevant to inflammation, blood clotting, bone formation, atherogenesis, regulation of lung barrier, and many others (Oskolkova and Bochkov 2020; Zhivaki and Kagan 2021; Di Gioia and Zanoni 2021; Karki and Birukov 2021; O’Donnell et al. 2019). Among several biological activities of OxPLs in endothelial cells, the induction of IL-8 is a prominent and pathologically relevant effect (Watson et al. 1997). In addition to its well-recognised role as a potent granulocytic chemoattractant, IL-8 demonstrates pleiotropic activities in cancer (Han et al. 2021), atherogenesis (Apostolakis et al. 2009), and angiogenesis (Ha et al. 2017; Liu et al. 2016). The pathological importance of this chemokine is illustrated by the fact that IL-8 and its receptors are targets of multiple investigative drugs, including both small molecule antagonists and neutralising antibodies ((Boyles et al. 2020) and references therein).

OxPLs induce IL-8 via complex and incompletely characterised mechanisms. Some cellular effects of OxPLs may be triggered by the impairment of proteostasis induced by electrophilic molecular species that are abundant in OxPAPC (Sonninen et al. 2020; Jyrkkänen et al. 2008; Gesslbauer et al. 2018). Proteome damage induces endoplasmic reticulum stress and unfolded protein response, which in turn trigger multiple intracellular events including the induction of IL-8 by OxPAPC (Gargalovic et al. 2006).

Impairment of proteostasis is a universal pathogenic event observed in a variety of diseases having very different ethiology, for example in neurodegenerative diseases (Sonninen et al. 2020). Stability of proteome is supported by several intracellular mechanisms including heat-shock proteins, which act as protective molecular chaperones in multiple disease conditions (Pilla et al. 2017). Several drug-like compounds are known that are capable of upregulating HSPs without inducing a cellular stress. They can act either alone or enhance the induction of HSPs in response to additional stress factors (Ohtsuka et al. 2005; Kuta et al. 2020). In this work, we show that structurally and mechanistically different HSP inducers inhibited the OxPAPC-induced upregulation of IL-8 in HUVECtert cells. Furthermore, chemical chaperones, which mimic the action of HSPs by direct protein stabilisation, also reduced the production of IL-8 in response to OxPAPC. Our results suggest that pharmacological stabilisation of proteome can protect cells from the pro-inflammatory effects of OxPLs mediated by the secretion of IL-8.

## Materials and methods

### Reagents

Plant-derived natural products and synthetic molecules with known or expected anti-inflammatory activity were purchased from different vendors. HA15 ((*N*-[4-[3-[[5-(dimethyl amino) naphthalen-1-yl]sulfonyl amino]phenyl]-1,3-thiazol-2-yl] acetamide, Calbiochem) was purchased from Sigma-Aldrich (St. Luis, MS). Geranylgeranyl acetone (GGA) and sodium salt of phenyl butyrate (PBA) were from Selleck Chemicals GmbH (Berlin, Germany). 2,3-Bis-(2-methoxy-4-nitro-5-sulfophenyl)-2*H*-tetrazolium-5-carboxanilide (XTT) and phenazine methosulfate (PMS) were from Thermo Fisher Scientific (Waltham, MA, USA) or (TCI, Tokyo, Japan), respectively. OxPAPC was prepared from 1-palmitoyl-2-arachidonoyl-*sn*-glycero-3-phosphocholine (Avanti Polar Lipids, Birmingham, AL, USA) and analysed as described (Watson et al. 1997). OxPAPC was stored as a chloroform solution at − 80 °C, and evaporated under the stream of argon before solubilisation in the cell culture medium.

### Cultivation and treatment of HUVECtert cells

HUVECtert cells were grown at 37 °C in 5% CO_2_ in medium M199 with Earl’s Salts (Gibco from Thermo Fisher Scientific) supplemented with 20% foetal bovine serum (Sigma-Aldrich), L-glutamine (Lonza, Basel, Switzerland), penicillin–streptomycin-fungizone (Lonza) and endothelial cell growth supplement containing heparin (ECGS/H) (PromoCell, Heidelberg, Germany) in Nunclon Delta 75 cm^2^ cell culture flasks (Thermo Fischer Scientific). After reaching 80% confluency, the cells were seeded into Nunclon Delta 96-well plates (Thermo Fisher Scientific). After 48 h, the full medium was aspirated and exchanged to 120 µl medium containing 1.25-fold concentrations of the test compounds dissolved in the medium M199 containing all supplements except ECGS/heparin and serum (assay medium). Stock solutions of the test compounds were prepared in DMSO; the final concentration of DMSO (0.1%) was equal in all wells. After 20-30 min, 30 µl of the 5-fold concentrated OxPAPC were added. To prepare the OxPAPC suspension, chloroform stock of OxPAPC was evaporated under the stream of argon with simultaneous vortexing to obtain a lipid film on the wall of a tube. After evaporation of chloroform, the pre-warmed assay medium was added and the tube vigorously vortexed for one minute, followed by warming for three minutes in a water bath at 37 °C and vortexing again for 30 s. The OxPAPC suspension was applied to the cells at 20 µg/ml end concentration, because preliminary experiments showed the highest IL-8 induction at this concentration.

Each well of the 96-well plate had an end volume of 150 µl. After 24 h of incubation of cells with compounds, 50 µl were removed for the IL-8 ELISA and after additional 48 h the XTT- and Hoechst assays were performed. Thus, all three assays were performed on the same samples and thus can be directly compared.

### IL-8 Sandwich ELISA

Detection of the CXCL8/IL-8 chemokine was performed using a sandwich ELISA (DuoSet, R&D Systems, Minneapolis, MN, USA) in 96-well MaxiSorp Plates (Thermo Fisher Scientific). A calibration curve was obtained using serial dilutions of the IL-8 standard from 0 to 2 ng/ml in the Reagent Diluent supplied with the kit. A two-component TMB substrate (Thermo Fisher Scientific) was used; after stopping the reaction, the optical density was measured at 450 nm. Calculations were performed using GraphPad Prism version 9.

### Metabolic activity (XTT) assay

After 72 h of the incubation with the compounds, the medium was removed and 50 µl of serum-free M199 were pipetted into each well. Then, 50 µl of the 2-fold concentrated XTT/PMS solution in the assay medium was added to the end concentration of 0.2 mg/ml XTT and 7.5 µg/ml PMS. After 2 h incubation at 37 °C in 5% CO_2_, the optical density at 450 nm was measured. As controls for the XTT and Hoechst assays, 0.1% Triton X-100 (Carl Roth, Karlsruhe, Germany) diluted in the assay medium was added for the same time period.

### Hoechst assay

Endothelial cells grow as adherent cells and therefore dead cells are lost from the surface. The method determines toxicity by counting nuclei of cells remaining on plastic after incubation with test substances. Directly after the measurement of the XTT assay, 50 µl of the 3-fold concentrated Hoechst solution in the assay medium were added to obtain the final concentration of 1 µg/ml Hoechst 33342 (Thermo Fisher Scientific). After 60 min, the stained nuclei were counted using an image analysis module in the PerkinElmer EnSight plate reader. The data were transferred to GraphPad Prism for further calculations and statistical tests. Triton X-100 treated cells served as a control for toxicity.

### Real-time PCR

After the stimulation of cells in 12-well Nunc plates for 4 h, the medium was removed, and RNAzol (Molecular Research Center, OH, USA) was added. Total RNA was isolated according to the manufacturer’s instructions. Concentrations of RNA were determined using DeNovix DS-11 + nanodrop (DeNovix, Wilmington, DE). Transcription of mRNA (900 ng) into cDNA was performed using FastGene Scriptase (Nippon Genetics, Dueren, Germany) and oligo-dT(16) (Applied Biosystems, CA, USA). RT-PCR was performed on a StepOnePlus instrument (Applied Biosystems, Waltham, MA, USA) using SybrGreen reagent (Nippon Genetics) and specific primers: β2-microglobulin (β2M) (Oskolkova et al. 2010), HSPA1 (NM_005346, Qiagen, Venlo, The Netherlands), HSPA6 (NM_002155, Qiagen), and IL-8 (Oskolkova et al. 2010). Real-time PCR was performed as described (Oskolkova et al. 2017). The expression level of target genes was normalized to β2M (Pfaffl 2001).

### Calculations and statistical tests

Statistical analysis was performed using the GraphPad Prism version 9. The data received from the ELISA measurements were re-calculated into IL-8 concentrations using calibration curves and the data interpolation (four parameter logistic equation) for each 96-well plate. The graphs present means and standard deviations of parallel measurements. The statistical significance has been estimated using a one-way ANOVA test followed by a post hoc Dunnett’s test for the comparison of all values to one control value. A *p*-value below 0.05 was considered as significant. ***p < 0.001.

## Results

### Plant-derived natural products inhibit the OxPAPC-stimulated production of interleukin-8 in endothelial cells

**Fig. 1:**
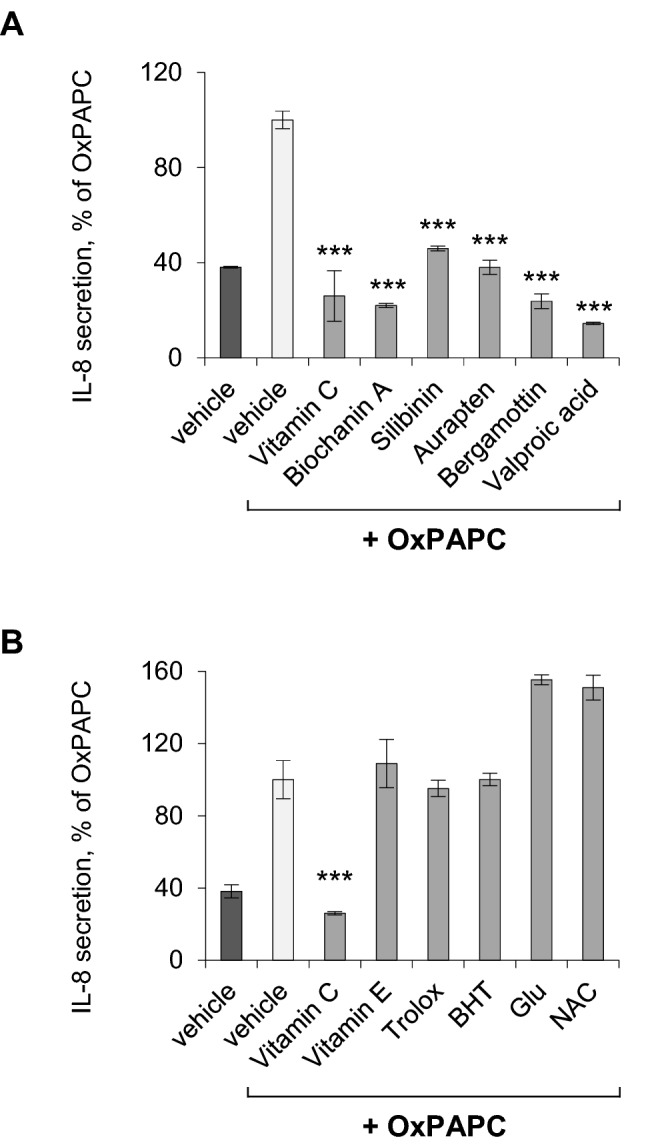
**Selected small molecules inhibit the secretion of IL-8 induced by OxPAPC via mechanisms independent of antioxidant activity.** HUVECtert cells were incubated with OxPAPC (50 µg/ml) for 5 h in the absence or presence of a broad panel of > 150 individual natural products and synthetic drug-like molecules, which were obtained from different commercial sources. The substances were added 30 min before OxPAPC. If a substance demonstrated a toxicity, its concentration in the next experiments was reduced to sort out substances acting via non-specific cell toxicity. In the presented figure, the compounds were used at 30 µM, except auraptene (5 µM) and valproic acid (500 µM). The data are representative of 2–5 independent experiments each performed in triplicates; the elevation of IL-8 induced by OxPAPC + vehicle (no inhibitors) was taken as 100%. Panel A presents active compounds that inhibited the OxPAPC-induced secretion of IL-8. Panel B shows that with the exception of vitamin C, several antioxidants and electrophile scavengers did not inhibit the induction of IL-8. *BHT* butylated hydroxytoluene, *Glu* glutathione, *NAC* N-acetylcysteine

Animal models show that OxPLs may play a causative role in the pathogenesis of different conditions accompanied by inflammatory reactions (Que et al. 2018). Identification of drug-like compounds inhibiting the production of inflammatory cytokines in response to OxPLs is an obligatory step in developing pharmacological therapy for neutralisation of OxPLs. In this work, we tested anti-inflammatory natural products and structurally related compounds for their ability to inhibit the OxPAPC-induced secretion of IL-8, which is an inflammatory mediator induced by OxPLs (Watson et al. 1997). The experiments were performed on HUVEC cells, which are an economical alternative to arterial endothelial cells thus allowing testing of multiple compounds. Data from our and other laboratories have shown that OxPLs trigger in HUVECs inflammatory reactions that are very similar to those induced in arterial cells, including the upregulation of IL-8 (Watson et al. 1997; Oskolkova et al. 2010). Six out of > 150 tested compounds at non-toxic concentrations reproducibly inhibited the induction of IL-8 protein by OxPAPC in endothelial HUVECtert cells (Fig. 1A). Some of the active compounds, for example vitamin C, have prominent antioxidant properties, while others, like valproic acid, are not antioxidants. On the other hand, several active antioxidants (vitamin E, Trolox, butylated hydroxytoluene) did not inhibit the induction of IL-8 by OxPAPC (Fig. 1B). Furthermore, N-acetylcysteine and glutamine, which contain soft and hard nucleophilic groups that can react with different electrophilic groups in OxPAPC, were also not inhibitory (Fig. 1B). Taken together, these findings suggest that antioxidant or carbonyl-trapping properties play a minor or no role in the anti-inflammatory activity of selected substances.

### Natural products potentiate the OxPAPC-induced transcription of heat-shock proteins

**Fig. 2:**
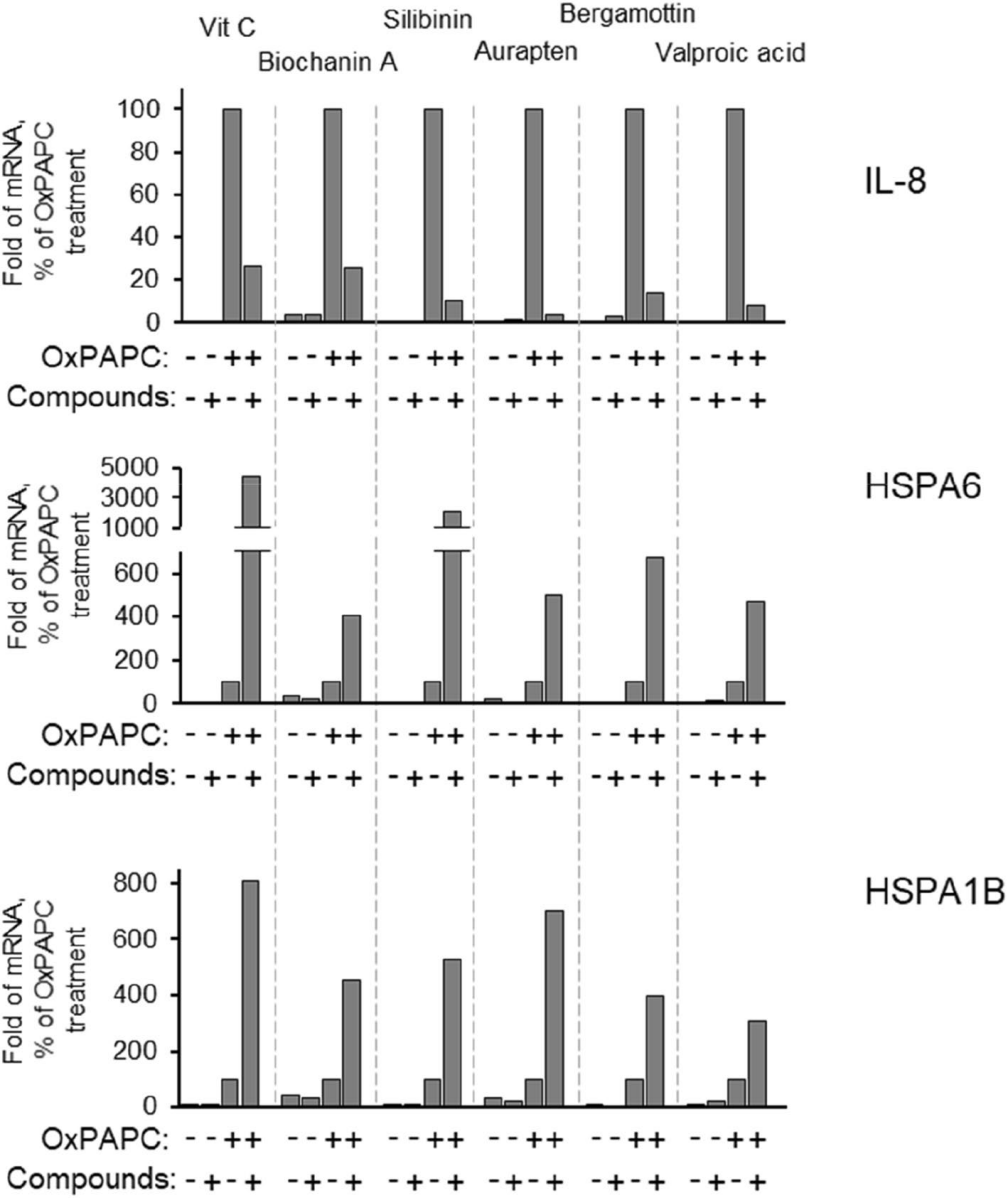
** Selected small molecules inhibit the OxPAPC-induced IL-8 mRNA elevation, but potentiate the OxPAPC-induced upregulation of** ***HSPA6*** **and** ***HSPA1B*** **genes**. HUVECtert cells stimulated with OxPAPC (50 μg/ml, 5 h) with or without six small molecules that were selected as describe in the Fig. 1. Identically treated samples were combined, analysed by real-time PCR and the values normalised to the β2-microglobulin mRNA levels

We further analysed effects of selected natural products on expression of a panel of genes induced by OxPAPC (*VEGFA*, *ATF3*, *PTGS2*, *HBEGF*, *ADM1*, *IL8* (*CXCL8*), *OSGIN1*, *HSPA6*, *NQO1*, *CXCL1*, *GCLM*, *KITLG*, *CXCL2*, *EGR1*, *HSPA1B*, *HMOX1*, *F3*, *CCL2*, *JUNB*, *VIP*, *TRIB3*). The genes were selected based on our microarray and real-time PCR data showing their reproducible induction by OxPAPC in HUVECs (not shown). Many of these OxPAPC-inducible genes are involved in proteostatic cell stress (heat shock, electrophilic stress, endoplasmic reticulum stress) and inflammation. Individual natural products demonstrated variable effects on different OxPAPC-induced genes. However, a few genes were consistently regulated by all six compounds. One common effect was the inhibition of IL-8 mRNA induction by OxPAPC, while another consistent finding was the regulation of heat-shock proteins HSPA1B and HSPA6 by all selected substances (Fig. 2). When added alone (i.e., without OxPAPC), the natural products had a minimal or no influence on the basal levels of HSPA1B and HSPA6 mRNAs. However, all compounds strongly enhanced the induction of HSPs by OxPAPC (Fig. 2). Thus, these natural products can be characterised as HSP co-inducers (Ohtsuka et al. 2005).

Previous studies have shown the importance of the unfolded protein response (UPR) in the induction of IL-8 by OxPLs (Gargalovic et al. 2006). These findings suggest that the upregulation of HSPs and the consequent stabilisation of proteome may be a mechanistic basis for the inhibitory action of natural products on the OxPAPC-induced upregulation of IL-8. To get an additional support for this hypothesis, we tested if chemically different pharmacological HSP inducers also inhibit the induction of the pro-inflammatory IL-8 by OxPAPC.

### Inducers of HSPs and chemical chaperones inhibit induction of IL-8 by OxPAPC

**Fig. 3:**
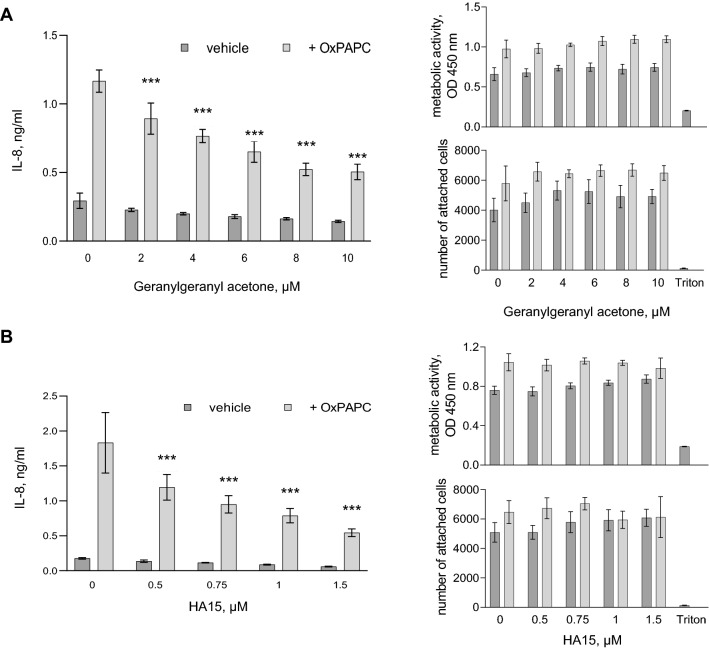
**Pharmacological HSP inducers geranylgeranyl acetone (GGA) and HA15 inhibit the IL-8 induction by OxPLs.** HUVECtert cells were pre-incubated in serum-free M199 (assay medium) for 20 min with the indicated concentrations of GGA (panel A) or HA15 (panel B) followed by addition of OxPAPC to 20 µg/ml. After 24 h, 50 µl aliquots were taken for IL-8 ELISA. The cells were further incubated in the rest of the medium (100 µl) for additional 48 h and then analysed by the XTT and Höchst cell toxicity assays

Geranylgeranyl acetone (GGA) is an acyclic isoprenoid compound characterised by cyto-protective properties based on its ability to upregulate HSPs in vitro and in vivo (Zeng et al. 2018). Importantly, in contrast to OxPAPC, GGA does not induce proteostatic stress. GGA and its derivatives prolong hyperphosphorylation and activation of HSF1 through incompletely understood mechanisms, which results in the enhanced transcription of HSPs (van Marion et al. 2019). We found that GGA dose-dependently inhibited the induction of IL-8 by OxPAPC (Fig. 3A, left panel). Importantly, two different cell assays confirmed that the effect was not due to the toxicity of GGA (right panels).

Another tested drug was HA15, an experimental anti-tumour thiazole benzene sulphonamide compound (Cerezo et al. 2016). HA15 inhibits the ATP-ase activity of GRP78 and thus mimics ER stress and triggers a low-grade physiological unfolded protein response (UPR) (Cerezo and Rocchi 2017). The UPR upregulates chaperones and other proteins involved in proteostasis through its IRE1α–XBP1 and ATF6 arms (He et al. 2010; Wang and Kaufman 2016). We found that HA15 significantly inhibited the induction of IL-8 in the OxPAPC-treated cells and was not toxic at the tested concentrations (Fig. 3B).

**Fig. 4:**
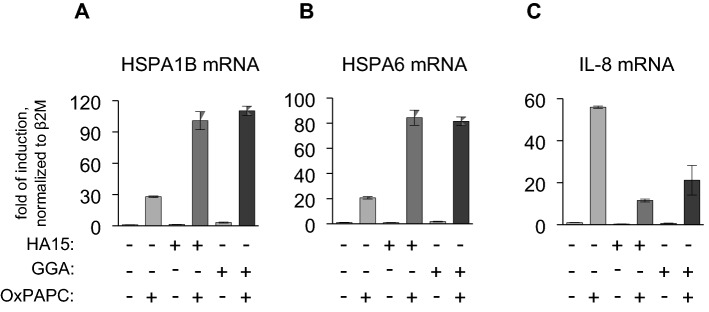
**Heat shock protein inducers geranylgeranyl acetone (GGA) and HA15 potentiate the expression of HSPA1B and HSPA6 induced by OxPAPC.** HUVECtert cells were treated in serum-free M199 (assay medium) with 20 µg/ml OxPAPC without or with GGA (10 µM) or HA15 (1.5 µM) for 5 h. GGA and HA15 were added 20 min before OxPAPC. Each combination of drugs and OxPAPC was analysed in sextuplicates. Samples were pooled and analysed for the expression of HSPA1B (**A**), HSPA6 (**B**), and IL-8 (**C**) mRNA by RT-PCR as described in the “Methods”. The SD values (**A** and **B**) correspond to 3 analytical replicates. Note the opposite regulation of HSPs vs IL-8 by HSP inducers

Analysis of the mRNA expression of two inducible HSPs—HSPA1B and HSPA6—showed that similarly to the natural products described above, both GGA and HA15 had no or only minimal effect on the basal levels of these mRNAs, but strongly amplified their induction by OxPAPC (Fig. 4A and B). This type of the pharmacological interaction may be classified as a potentiation, and compounds—as HSP co-inducers (Ohtsuka et al. 2005). Importantly, the pharmacological HSP co-inducers alone do not trigger proteostatic stress. In accordance with IL-8 protein expression data, GGA and HA15 inhibited the OxPAPC-induced elevation of IL-8 mRNA (Fig. 4C).

**Fig. 5:**
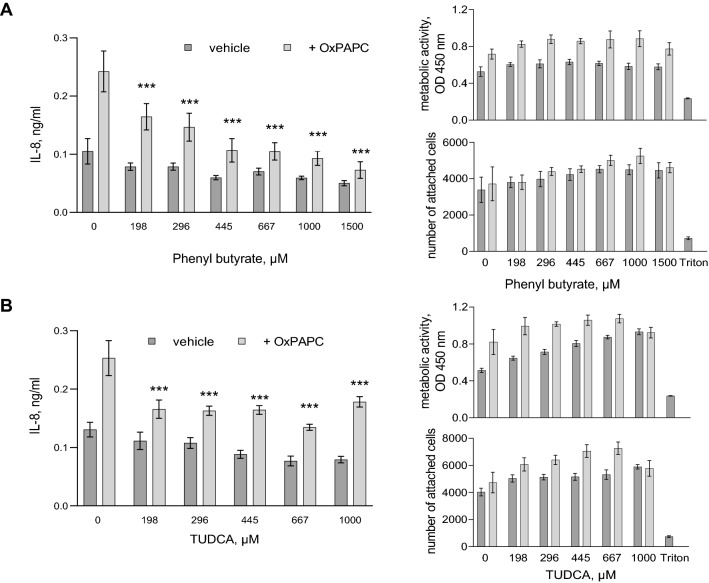
**The chemical chaperones PBA and TUDCA inhibit the IL-8 induction by OxPAPC.** Cells were pre-incubated (20 min) in serum-free M199 (assay medium) with the indicated concentrations of PBA (panel **A**) or TUDCA (panel **B**), followed by addition of 20 µg/ml OxPAPC. After 24 h, 50 µl aliquots were taken for IL-8 ELISA. The cells were further incubated in the rest of the medium (100 µl) for additional 48 h and then analysed by the XTT and Höchst cell toxicity assays

HSPs may have additional functions that are not directly linked with their chaperone function, e.g., receptor functions (Birukova et al. 2014) or an activation of pattern-recognition receptors (Pockley 2003). In order to get evidence that the inhibitory effect of HSPs on the IL-8 induction was linked to their role in proteostasis, we applied an HSP-independent approach based on protein-stabilising chemical chaperones PBA and TUDCA. In contrast to HSP inducers, these compounds can directly stabilise protein structures and thus mimic action of HSPs (Cortez and Sim 2014). We found that both chemical chaperones reduced the OxPAPC-induced production of IL-8 and that this action was not due to the toxicity of the compounds (Fig. 5).

To summarise, our experimental data show that in addition to natural products that upregulate HSPs, also two mechanistically different HSP inducers and two chemical chaperones inhibited the OxPAPC-induced elevation of IL-8. The data suggest that proteostatic stress plays an important role in the induction of IL-8 by OxPLs and that the protection of proteome by substances that either upregulate HSPs or mimic their action potentially can neutralise this deleterious effect.

## Discussion

The major finding of this work is a proof-of-principle that small drug-like molecules can prevent the induction of IL-8 by OxPAPC, a pro-inflammatory DAMP molecule accumulating during oxidative stress, which is characteristic of essentially every type of pathology. Importantly, the activity seems to be independent of either antioxidant or carbonyl-scavenging properties but is likely to result from the ability of the tested molecules to upregulate heat-shock proteins or mimic their proteostatic action.

We did not aim at characterising molecular mechanisms linking HSPs with pathways of the IL-8 induction; this will require significant effort in the follow-up projects. The most important result of this work is identification of “heat shock”-inducible proteome as a *pharmacological target* for the development of non-antioxidant drugs neutralising deleterious effects of lipid peroxidation products such as OxPLs. Such drugs are urgently needed because, on one hand, the pathogenic role of lipid peroxidation is well documented, but on the other hand, effective approaches for antioxidation and carbonyl trapping in patients are still missing.

Some of the compounds that demonstrated the anti-IL-8 activity in our experiments, for example valproic acid, phenyl butyric acid and geranylgeranyl acetone, are approved for treatment of human diseases. For example, valproic acid is widely used for the treatment of epilepsy, which is accompanied by the accumulation of IL-8 and other cytokines in the cerebrospinal fluid (Youn et al. 2013). Further studies are needed to test if the anti-inflammatory activity plays a role in the therapeutic action of these drugs. Another promising direction of research is generation of HSP inducers with better pharmacokinetics, for example derivatives of geranylgeranyl acetone (van Marion et al. 2019).

In summary, our findings suggest repurposing the HSP inducers and the chemical chaperones as a pharmacological approach for the neutralisation of IL-8-dependent effects of OxPLs. The data justify further detailed studies of proteostasis and its chemical protectors in models of pathology accompanied by oxidative stress and generation of OxPLs.

## Data Availability

Not applicable.

## Abbreviations

β2M: β2-Microglobulin
DAMPs: Danger-associated molecular patterns
ECGS: Endothelial cell growth supplement
GGA: Geranylgeranyl acetone
GRP78: Glucose-regulated protein 78
HA15: *N*-[4-[3-[[5-(Dimethyl amino) naphthalen-1-yl]sulfonyl amino]phenyl]-1,3-thiazol-2-yl] acetamide
HSP: Heat-shock protein
HUVECtert: Immortalised human umbilical vein endothelial cells
IL-8: Interleukin 8
OxPAPC: Oxidised 1-palmitoyl-2-arachidonoyl-*sn*-glycero-3-phosphocholine
OxPLs: Oxidised phospholipids
PBA: Phenyl butyrate sodium salt
PMS: Phenazine methosulfate
TUDCA: Taurourso deoxycholic acid
UPR: Unfolded protein response
XTT: 2,3-Bis-(2-methoxy-4-nitro-5-sulfophenyl)-2H-tetrazolium-5-carboxanilide
